# Leaching kinetics and mechanisms in efficient chlorination leaching of germanium-rich fume dust

**DOI:** 10.1039/d6ra01890d

**Published:** 2026-07-02

**Authors:** Enle Xu, Chenyu Zhang, Ying Sun, Xu Liu, Zhenyong Miao

**Affiliations:** a State Key Laboratory of Coking Coal Resources Green Exploitation, China University of Mining and Technology Xuzhou Jiangsu 221116 China zymiao@cumt.edu.cn; b School of Chemical Engineering and Technology, China University of Mining and Technology Xuzhou Jiangsu 221116 China; c National Engineering Research Center of Coal Preparation and Purification, China University of Mining and Technology Xuzhou Jiangsu 221116 China; d China Power Engineering Consulting Group Co., Ltd Beijing 100029 China

## Abstract

Chlorination distillation is a critical step in the efficient purification of germanium, as it directly influences the quality of the product. However, the current chlorination distillation system still suffers from constrained germanium leaching efficiency and excessive acid consumption, primarily mediated by leaching kinetics. This study establishes an apparent kinetic model for the chlorination leaching system of germanium-rich fume dust within the framework of thermodynamics. The results showed that the leaching efficiency of germanium was as high as 94.43%. The leaching process kinetics of germanium follows the Avrami equation, with all *n* values less than 0.5 and an apparent activation energy of 8.89 kJ mol^−1^, indicating a diffusion-controlled process associated with mass transfer limitations. Meanwhile, impurities were found to preferentially consume hydrochloric acid to reduce the leaching efficiency of germanium, especially at low acid concentrations. Density functional theory (DFT) and sequential chemical extraction analyses indicate that germanium liberation proceeds through proton-assisted cleavage of Ge–O bonds followed by chloride coordination to form Ge–Cl-containing Ge(iv) species, with the leached germanium present as GeCl_4_, while unextracted germanium remains in the residue as complex germanosilicate crystalline phases. The elucidation of the leaching kinetics and efficient release mechanisms of germanium provides a theoretical foundation for the optimization of the efficiency and selectivity of chlorination leaching, which are crucial for achieving green recovery of germanium resources.

## Abstract 2

Leaching kinetics and mechanistic pathways in the chlorination-efficient extraction of germanium-rich fume dust were clarified through integrated kinetic modeling, surface analysis and theoretical calculations.

## Graphical abstract

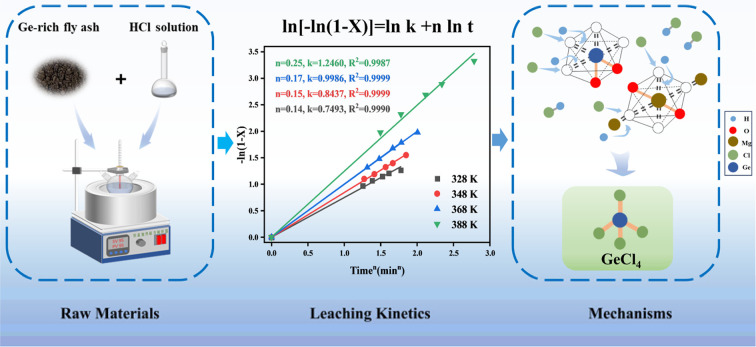

## 1 Introduction

Germanium, a strategically scarce and dispersed metal, plays an irreplaceable role in high-tech industries such as defence and aerospace.^1–4^ China holds 3526 tons of germanium reserves, accounting for 41% of the global total, and produces nearly 70% of the world's germanium, solidifying its position as the largest global producer and exporter.^5^

The global germanium supply system exhibits a ternary structure: germanium-associated lignite deposits,^6,7^ lead–zinc smelting byproducts,^8^ and recycled electronic waste resources.^9,10^ Current methods for extracting germanium from germanium-bearing materials employ distinct front-end processes,^11–13^ such as acid/alkali leaching,^14,15^ high-temperature volatilization,^16^ microbial oxidation/reduction,^17^ or organic solvent extraction^18^ to obtain germanium-rich materials. Given the relatively low germanium content in these intermediate products, a subsequent enrichment step is typically necessary. As the key technology of germanium enrichment, chlorination distillation,^8^ firstly achieves efficient leaching of germanium through chlorination reaction, and then separates and purifies germanium by distillation under atmospheric pressure. The combination of chlorination leaching and distillation significantly improves the recovery and purity of germanium.

Chlorination leaching technology has received great attention in germanium metallurgy. Zhu *et al.*^19^ developed the chlorination roasting-acid leaching combined process, and the recovery of germanium reached 90% under the condition of hydrochloric acid concentration of 10 mol L^−1^. Zhang *et al.*^20,21^ innovated the vacuum metallurgy-chlorination distillation coupling technology to achieve a total recovery rate of 91.88% of germanium at 8 mol L^−1^ hydrochloric acid from fly ash. Bo *et al.*^22^ used gravity separation combined with low-temperature sintering and chlorine distillation to enrich germanium in lignite by a factor of 10.6, recovering 88.8% germanium at a hydrochloric acid concentration of 7 mol L^−1^ and a liquid–solid ratio of 30 : 1. Significant progress has been achieved in germanium chlorination leaching technologies.

Leaching kinetics is the theoretical basis for improving extraction efficiency and reducing energy consumption, which directly affects the final leaching rate of the target product.^14^ Rao *et al.*^23^ studied the kinetics of germanium concentrate under hydrochloric acid leaching and found that the leaching of germanium was controlled by chemical reaction and diffusion.

Song *et al.*^24^ studied the kinetics of germanium-containing materials under sulfuric acid leaching and found that the leaching of germanium was controlled by diffusion. Although extensive research has been conducted on germanium leaching kinetics, most existing studies have primarily focused on relatively simple systems such as germanium concentrates or high-grade materials, where impurity effects are minimal and phase compositions remain well-defined. In contrast, germanium-rich fume dust typically contains less than 1% germanium and is characterized by high impurity contents together with complex phase associations involving germanosilicate phases. These characteristics modify both the effective acidic environment and mass transfer dynamics, which in turn significantly influence leaching efficiency and kinetic parameters.

Against this background, the present study focuses on the chlorination leaching of germanium from germanium-rich fume dust in hydrochloric acid media. By integrating kinetic modeling with microstructural characterization and density functional theory (DFT) calculations, this work systematically examines the leaching behaviour and release mechanisms of germanium within complex fume dust systems, with particular emphasis on impurity interactions and phase structural evolution during the chlorination leaching process. This study is expected to provide a theoretical basis and technical support for the efficient extraction of germanium from compositionally complex germanium-bearing resources, thereby contributing to the sustainable recovery of germanium resources.

## 2 Experiments

### 2.1 Materials

The raw materials used in this study were obtained from germanium-rich fume dust from the combustion of germanium-bearing lignite in Inner Mongolia. Hydrochloric acid (HCl, 36–38 wt%) for the preparation of the leaching agent was purchased from Sinopharm Chemical Reagent Co., Ltd, Shanghai, China.

### 2.2 Experimental conditions

**Fig. 1:**
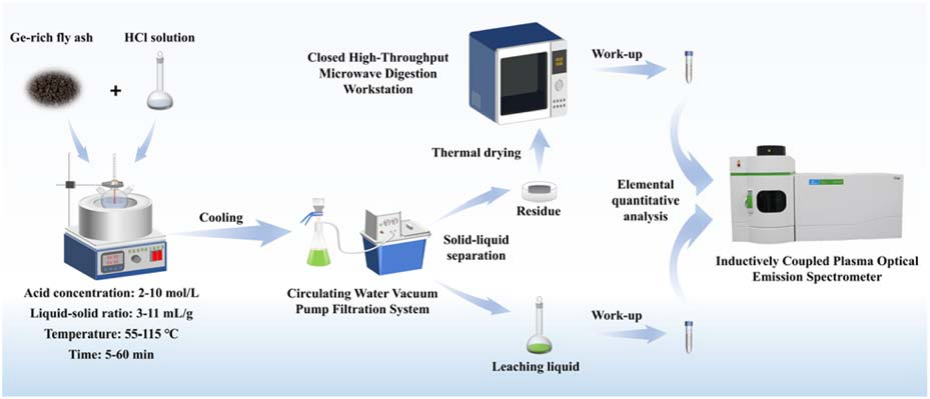
Flow chart of leaching experiment.

The effects of key process parameters including initial hydrochloric acid concentration, liquid-to-solid ratio, leaching temperature, and reaction time on the leaching kinetics of germanium from germanium-rich fume dust were investigated in this study. The experimental conditions are detailed in Table 1, with the workflow illustrated in Fig. 1. The procedure comprised the following steps:

**Table 1:** Experimental conditions and range of variations

Experimental conditions	Acid concentration	Liquid-solid ratio	Leach temperature	Leach time
Range of variations	2.0–10.0 mol L^−1^	3–11 mL g^−1^	328–388 K	5–60 min

Firstly, a hydrochloric acid leaching solution was prepared at the desired concentration and thoroughly mixed. Subsequently, the solution was transferred to a round-bottom flask, heated to the target temperature, and kept tightly sealed to minimize hydrochloric acid volatilization during heating. Finally, 5.00 g of germanium-rich fume dust was added to the flask to initiate the leaching process. Upon completion, the slurry was cooled, vacuum-filtered, and washed three times to separate the leachate from residues. The residual solids were vacuum-dried at 343 K until constant weight was achieved. The germanium contents in the leachate and residue were determined by ICP-OES, and the leaching efficiency was calculated using eqn (1). All leaching experiments in this study were performed in triplicate. The data presented in the text represent the mean values of three independent experiments, and error bars are shown in the corresponding figures.1
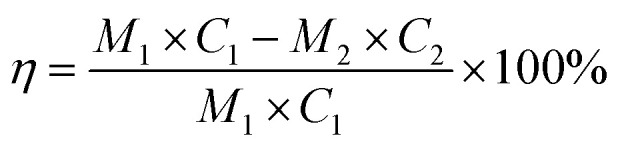


In eqn (1), *η* represents the germanium leaching efficiency (%), *M*_1_ and *M*_2_ are the mass of raw material and leaching residue respectively (g), *C*_1_ and *C*_2_ are the content of germanium in raw material and leaching residue respectively (%).

### 2.3 Characterization methods

Quantitative phase analysis of germanium-rich fume dust was performed using an X-ray diffractometer (XRD, D8 ADVANCE), complemented by semi-quantitative elemental scanning *via* X-ray fluorescence spectroscopy (XRF, S8 TIGER). Particle size distribution analysis was performed by a laser particle sizer (MASTERSZIER 3000). Microstructural characteristics and elemental distribution patterns were elucidated through field-emission scanning electron microscopy (FSEM, TESCAN MAIA3 LMH) coupled with energy-dispersive spectroscopy. Electron probe microanalyzer (EPMA-8050G) was used to perform quantitative elemental analysis of micro-regions in the fume dust. X-ray photoelectron spectrometer (XPS, ESCALAB 250Xi) was utilized to analyse the chemical states of elements in both fume dust and leachate. For liquid samples, the solution was drop-cast onto a silicon substrate and dried at 70 °C prior to XPS analysis. Density functional theory (DFT) simulations were employed to construct germanium-containing molecular models and calculate bond energy’s of critical Ge chemical bonds. Germanium quantification in fume dust, leaching residues, and leachates was executed using inductively coupled plasma optical emission spectrometry (ICP-OES, Optima 8300). Prior to analysis, standard solutions of known concentrations were utilized to establish calibration curves for each analysed element. Only calibration curves with correlation coefficients (*R*^2^) greater than 0.999 were accepted for subsequent measurements. Each sample was measured twice by ICP-OES, and the reported values represent the average of the two measurements to ensure analytical reliability.

## 3 Results and discussion

### 3.1 Material analysis

**Fig. 2:**
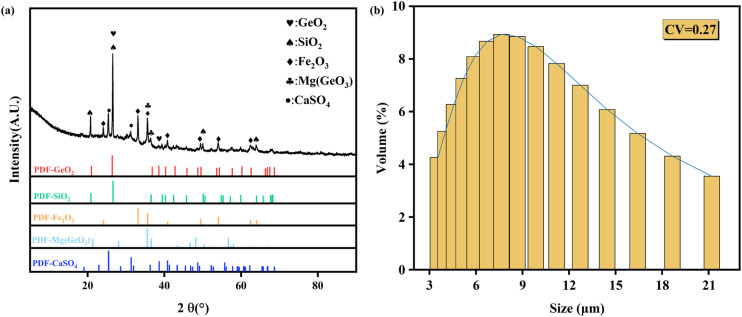
Characterization of the fume dust: (a) XRD patterns; (b) particle size distribution.

**Fig. 3:**
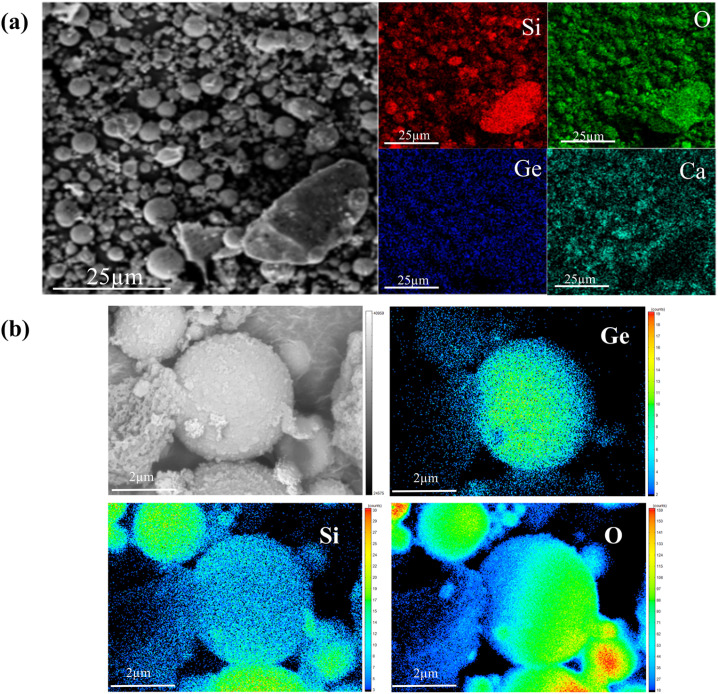
Microstructure diagram of germanium-rich fume dust (a) FSEM-EDS morphology and elemental mapping; (b) EPMA elemental mapping.

Before the XRD phase analysis, the germanium-rich fume dust was treated in an air atmosphere at 873 K for 6 h to burn the carbon in the fume dust and increase the germanium content in the fume dust. Fig. 2a shows the XRD patterns of fume dust, the diffraction peaks of which are readily indexed to the SiO_2_, GeO_2_, and Mg(GeO_3_). The particle size distribution of fume dust was in the range of 3.5–21.5 µm, as shown in Fig. 2b. Quantitative ICP-OES analysis (Table 2) revealed that the primary elements were Si, Fe, Al, Ca and Mg, with Ge present as a trace element accounting for 0.53% by mass. SEM-EDS results (Fig. 3a) show that the main elements are Ca and Ge, and the distribution of Ge elements is significantly spatially correlated with Si and O. EPMA surface analysis (Fig. 3b), indicates that Ge, O and Si have strong compatibility, which, combined with the shift of the characteristic peaks of SiO_2_ to lower angles in the XRD patterns, confirms that Ge^4+^ replaces Si^4+^ in the form of isomorphism, *i.e.* Ge–O–Si is formed in the fume dust. Moreover, fume dust presents sub-micron irregular small particles and regular spherical large particles.

**Table 2:** Main chemical composition of fume dust

Element	Si	Fe	Al	Ca	Mg	Ge
Mass fraction/%	17.31	11.66	8.86	8.38	2.28	0.53

**Fig. 4:**
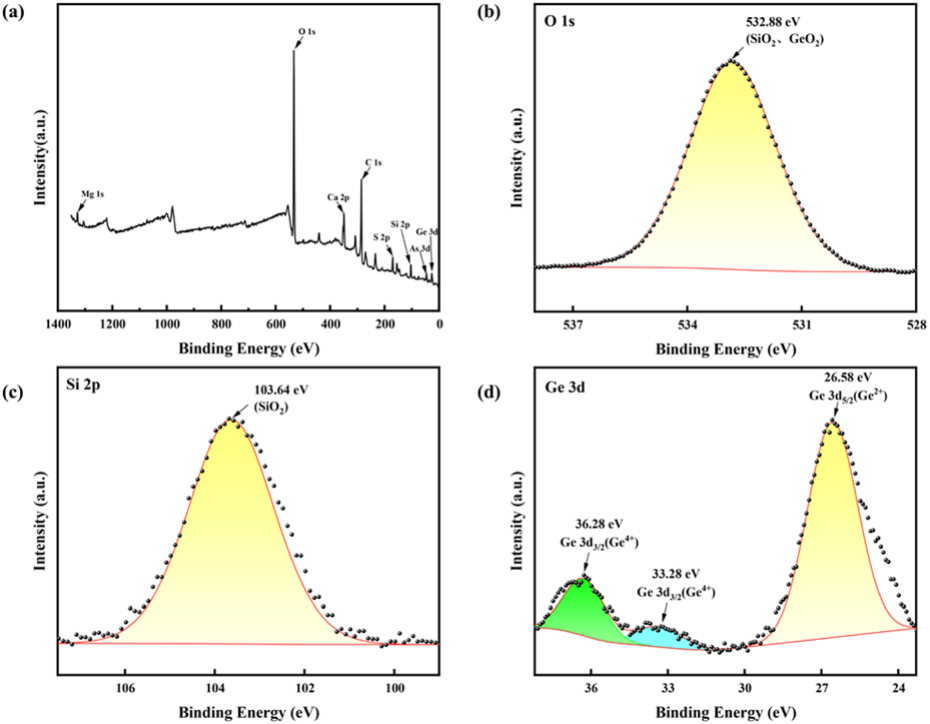
X-ray electron spectral analysis (a) full spectrum analysis; (b) O 1s fine spectrum; (c) Si 2p fine spectrum; (d) Ge 3d fine spectrum.

The XPS wide-scan survey spectrum of the raw material (Fig. 4) revealed characteristic peaks corresponding to O, Si, C, Ca, Mg, S, Ge, and As. High-resolution narrow-scan analysis of the Si 2p, O 1s, and Ge 3d orbital binding energies was performed using peak deconvolution and fitting. The results showed that the O 1s orbital binding energy peak at 532.88 eV corresponds to the typical coordination environment of metal–oxygen bonds. The Si 2p orbital binding energy peak at 103.64 eV aligns with the tetrahedral coordination characteristic of Si–O bonds.^25^ The characteristic peak of GeO_2_ appeared at 33.28 eV, while a subset of peaks exhibited a chemical shift toward a higher binding energy of 36.28 eV.^26,27^ Peak fitting analysis confirmed that both Ge and Si exist in the oxide lattice as tetrahedrally coordinated cationic species. The observed shift in the Ge 3d binding energy originates from the formation of Ge–O–Si bonds. The strong electronegativity of Ge–O–Si bonds reduces the electron cloud density around Ge atoms, leading to an increase in binding energy in accordance with Koopmans' theorem.^28^

Based on the comprehensive characterization results, germanium in this germanium-rich fume dust primarily exists in germanium oxide and germanate phase.

### 3.2 Thermodynamic analysis of leaching process

Given that germanium in germanium-rich fume dust primarily exists as germanium dioxide (GeO_2_) and magnesium germanate (Mg(GeO_3_)), the following chemical reactions are likely to occur in concentrated hydrochloric acid solution (Table 3):

**Table 3:** Possible reactions during leaching processes

Reactions	
GeO_2_ + 4 HCl = GeCl_4_ + 2 H_2_O	(R1)
GeO_2_*2MgO + 4 HCl = 2 MgCl_2_ + Ge(OH)_4_	(R2)
Ge(OH)_4_ + 4 HCl = GeCl_4_ + 4 H_2_O	(R3)
GeCl_4_ + 2 H_2_O = GeO_2_ + 4 HCl	(R4)
Fe_2_O_3_ + 6 HCl = 2 FeCl_3_ + 3 H_2_O	(R5)
As_2_O_3_ + 6 HCl = 2 AsCl_3_ + 3 H_2_O	(R6)
Al_2_O_3_ + 6 HCl = 2 AlCl_3_ + 3 H_2_O	(R7)

**Fig. 5:**
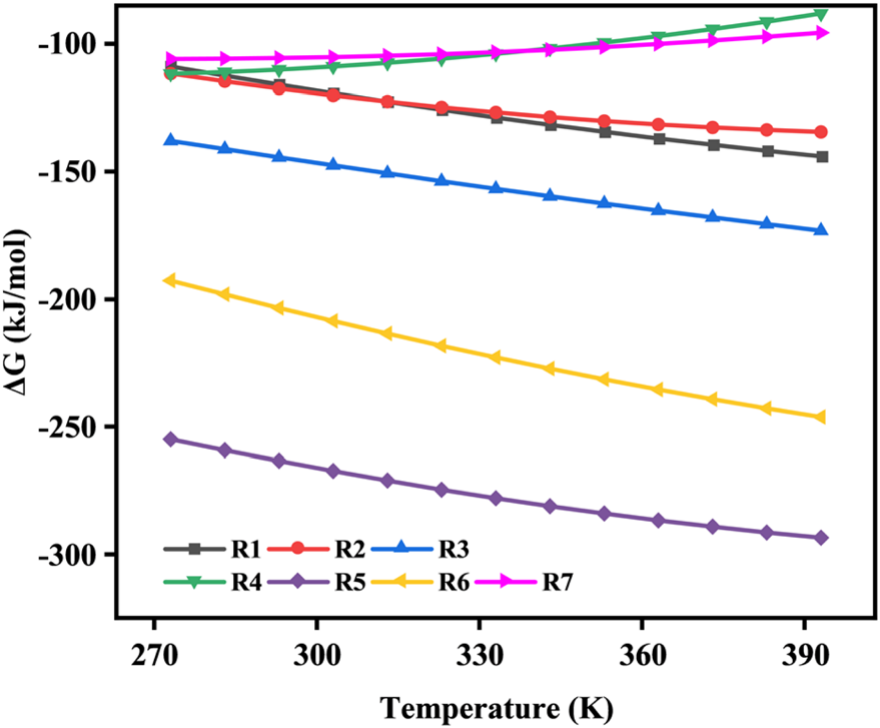
Thermodynamic calculations for each reaction in the standard state.

The Gibbs free energy (Δ*G*) of the aforementioned reactions at atmospheric pressure within the temperature range of 273–393 K was calculated using HSC Chemistry software. The relationship between Δ*G* and temperature for these reactions is illustrated in Fig. 5. Thermodynamic analysis revealed that all primary and secondary reactions in the chlorination system of germanium extraction from germanium-rich fume dust exhibited strongly negative Δ*G* values, confirming the high thermodynamic spontaneity of germanium chlorination across phases.

Thermodynamic analysis reveals that as temperature increases, the Δ*G* of GeCl_4_ formation becomes significantly lower than that of its hydrolysis, indicating a substantial thermodynamic advantage favoring chlorination over hydrolysis. This drives the reaction toward GeCl_4_ production.

### 3.3 Effect of leaching conditions

This section systematically investigates hydrochloric acid leaching of germanium from germanium-rich fume dust under standard conditions. Using hydrochloric acid as the leaching agent, the effects of various process parameters on germanium extraction efficiency were examined.

#### 3.3.1 Effect of initial concentration of hydrochloric acid

The effects of initial hydrochloric acid concentration on germanium leaching efficiency, mass loss efficiency, main impurities leaching efficiency and acid consumption were investigated under controlled conditions: liquid-to-solid ratio of 7 mL g^−1^, leaching temperature at 388 K, and leaching duration of 30 minutes.

**Fig. 6:**
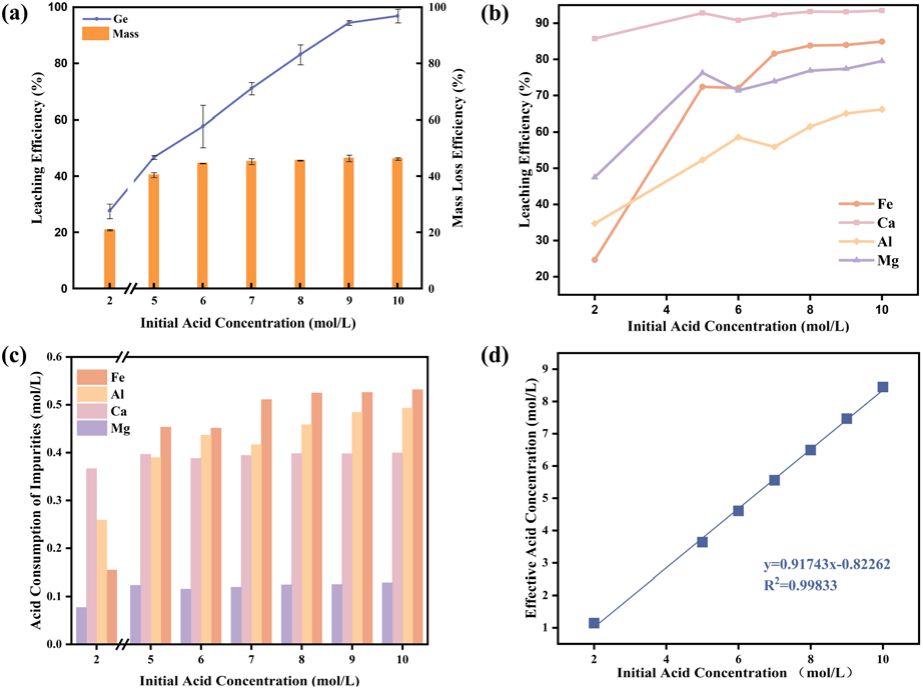
Effects of initial acid concentration on leaching behaviors (a) mass loss efficiency and germanium leaching efficiency; (b) leaching efficiency of major impurities; (c) acid consumption profiles of major impurities; (d) relationship between initial and effective acid concentrations for germanium leaching.

As illustrated by Fig. 6a and b, the effects of initial acid concentration on germanium leaching efficiency, major impurities leaching efficiency, and mass loss efficiency are significant. An increase in hydrochloric acid concentration from 2 to 10 mol L^−1^ resulted in a corresponding rise in the efficiency of germanium leaching from 27.64% to 96.88%. Concurrently, the iron leaching efficiency increased from 24.68% to 84.93%, the aluminum leaching efficiency grew from 34.73% to 66.16%, the calcium leaching efficiency improved from 85.75% to 93.50%, the magnesium leaching efficiency advanced from 47.52% to 79.51%, and the mass loss efficiency escalated from 20.98% to 46.24%. The germanium leaching efficiency demonstrated an initial gradual increase, followed by a decelerated growth pattern. Conversely, the impurity leaching efficiency and mass loss efficiency exhibited rapid initial growth, which eventually stabilized.

The calculation of acid consumption was derived from the metal content and leaching efficiency in accordance with their dissolution stoichiometry. As illustrated in Fig. 6c, the consumption of acid by impurities is observed to vary with differing initial acid concentrations. An increase in acid concentration from 2 to 10 mol L^−1^ resulted in an escalation in acid consumption for iron from 0.1544 to 0.5315 mol L^−1^, for aluminium from 0.2587 to 0.4928 mol L^−1^, for calcium from 0.3661 to 0.3992 mol L^−1^, and for magnesium from 0.0768 to 0.1285 mol L^−1^. Fig. 6d demonstrates that during the leaching process of germanium, the effective acid concentration increases linearly with the initial acid concentration. An increased effective acid concentration enhances hydrogen ion availability, which promotes germanium leaching efficiency. Conversely, preferential acid consumption by impurities diminishes the effective acid available for reaction, particularly at low acid concentrations, thereby inhibiting germanium leaching efficiency.

In the 2–5 mol L^−1^ range, the germanium leaching rate remains suboptimal at 46.76%. The constrained leaching performance was primarily attributable to two factors. Firstly, substantial dissolution of metal oxides (*e.g.* Fe_2_O_3_, CaO, and Al_2_O_3_) at low acid concentrations consumed most available acid, leaving insufficient acid concentration for effective germanium dissolution. Secondly, significant hydrolysis of GeCl_4_ occurred at acid concentrations below 5.0 mol L^−1^, generating insoluble GeO_2_ that remained in the residue.

In the 5–9 mol L^−1^ range, germanium leaching efficiency experienced a rapid enhancement (from 46.76% to 94.43%). The reason may be that elevated acid concentrations progressively depleted leachable components within the raw material, resulting in stabilized mass loss percentages, major impurity leaching efficiencies, and acid consumption rates. Simultaneously, the effective acid concentration available for germanium leaching increased substantially. The high-acid environment simultaneously suppressed hydrolysis reactions while facilitating GeCl_4_ formation, thereby enhancing germanium dissolution kinetics. Within the 9–10 mol L^−1^ acid concentration range, germanium leaching efficiency exhibited diminishing returns (increasing from 94.43% to 96.88%). This marginal improvement occurred due to near-complete extraction of accessible germanium, with residual germanium existing as chemically stable germanosilicate compounds demonstrating significant resistance to further acid leaching.

In conditions of less than 9 mol L^−1^, the major impurities had already been almost completely leached, and the mass loss percentage of the raw material had stabilized. Nonetheless, there was a further rapid increase in the efficiency of germanium leaching. This enabled the acid solution environment to focus more effectively on germanium leaching, providing ideal conditions for studying the kinetics of germanium leaching.

#### 3.3.2 Effect of liquid-to-solid ratio

The leaching experiments were conducted using 9 mol L^−1^ concentrated hydrochloric acid at 388 K to investigate the influence of liquid-to-solid ratio on germanium extraction efficiency.

**Fig. 7:**
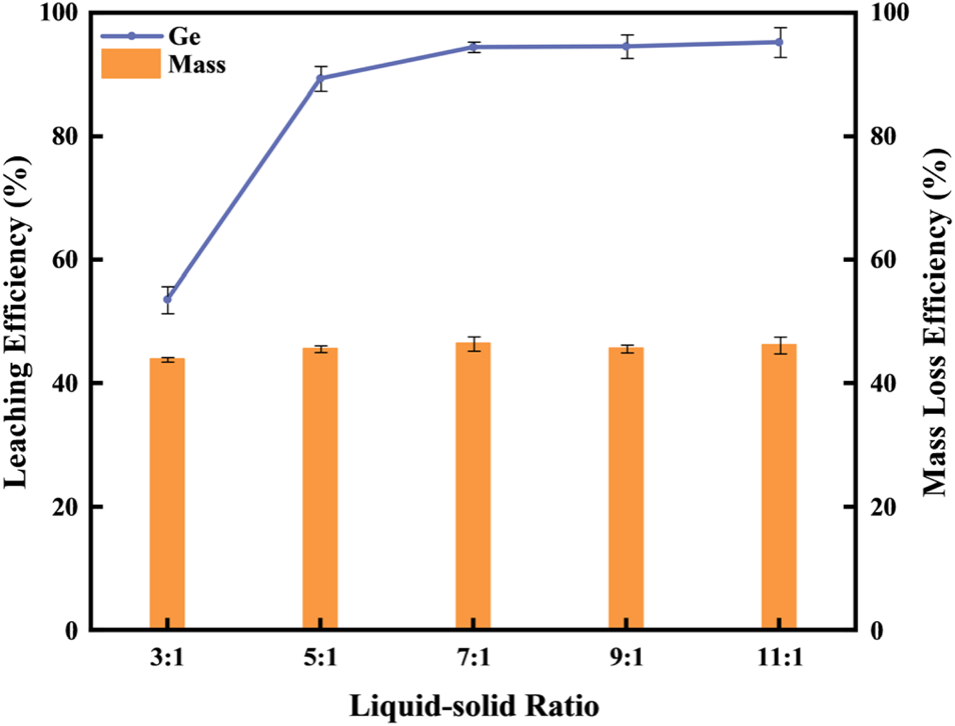
Effect of liquid–solid ratio on mass loss efficiency and germanium leaching efficiency.

Fig. 7 shows the effect of the liquid-to-solid (*L*/*S*) ratio on the efficiency of germanium leaching. The extraction efficiency increased from 53.59% to 95.21% as *L*/*S* ratio rose from 3 to 11 mL g^−1^, displaying an initial rapid growth followed by gradual stabilization. In the 3–5 mL g^−1^*L*/*S* range, germanium extraction efficiency surged from 53.59% to 89.32%. This substantial improvement stems from enhanced HCl availability at higher *L*/*S* ratios, which facilitates GeCl_4_ formation through improved reactant dissolution. Between 5–7 mL g^−1^, the growth efficiency decelerated (89.32% to 94.43%). Further increasing to 11 mL g^−1^ showed negligible improvement, with efficiency stabilizing around 95%. This plateau indicates optimal mass transfer conditions have been achieved. These results demonstrate that increasing *L*/*S* ratio effectively enhances extraction efficiency up to 7 mL g^−1^, beyond which additional increases yield diminishing returns due to concentration gradient limitations. While elevated *L*/*S* ratios improve solid–liquid mixing and mass transfer, excessively high ratios reduce dissolved metal concentrations and increase operational costs through excessive reagent consumption.

The mass loss efficiency showed minimal variation (43.93% to 46.26%) across *L*/*S* ratios, as most components in fume dust were fully dissolved under initial high-acid/high-temperature conditions. This observation aligns with the stabilized germanium extraction pattern at higher *L*/*S* ratios, confirming complete matrix dissolution.

Acid concentration and liquid-to-solid ratio are critical parameters controlling leaching kinetics. Experimental data confirm that higher acid concentrations and liquid-to-solid ratios significantly improve leaching performance, with GeCl_4_ becoming the dominant species in the leachate. However, excessively high HCl concentrations not only complicate downstream processes but also raise environmental concerns due to increased waste acidity. To balance efficiency and environmental impact, subsequent experiments adopted 9 mol L^−1^ HCl and a liquid-to-solid ratio of 7 mL g^−1^, optimizing germanium recovery while minimizing ecological risks.

#### 3.3.3 Effect of leaching time and temperature

The effects of leaching temperature and duration on germanium extraction efficiency were investigated using 9 mol L^−1^ HCl solution with a liquid-to-solid ratio of 7 mL g^−1^.

**Fig. 8:**
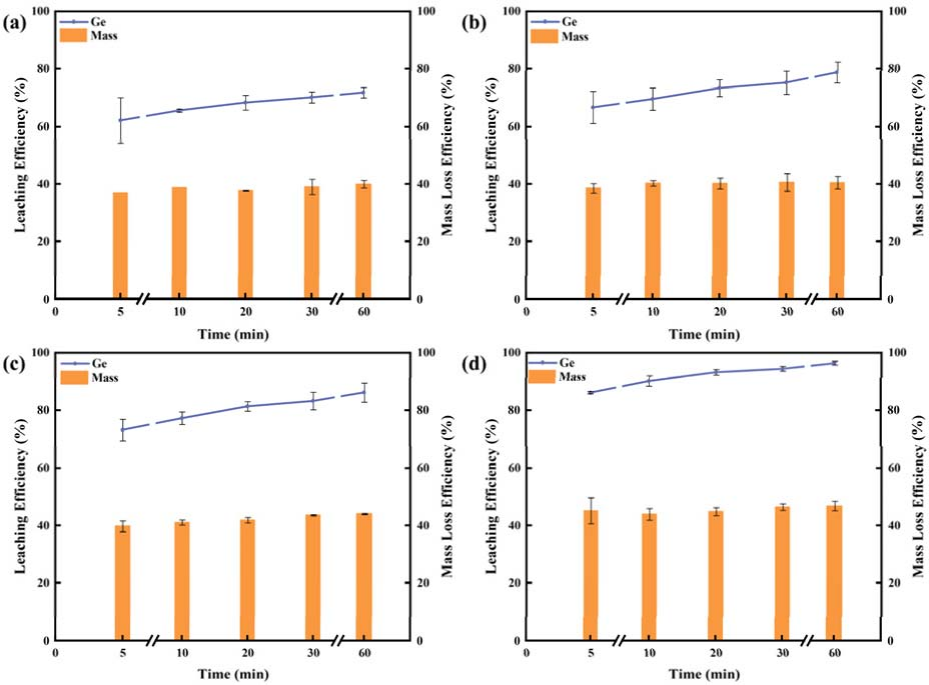
Effect of leaching time and temperature on mass loss efficiency and germanium leaching efficiency (a) 328 K; (b) 348 K; (c) 368 K; (d) 388 K.

Fig. 8 demonstrates that germanium leaching efficiency progressively increases with prolonged leaching time until reaching kinetic equilibrium. Under fixed 30 minutes leaching conditions, increasing temperature from 328 K to 388 K enhanced germanium extraction efficiency from 70.18% to 94.43%, indicating thermally activated reaction kinetics.

Mass loss efficiency exhibited parallel trends with germanium extraction, increasing from 37.04% to 46.87%. The stabilization of mass loss efficiency at 388 K, 30 min conditions corresponds to complete matrix dissolution, as confirmed by residual analysis in similar systems.

The experimental data reveal a synergistic effect between temperature and duration on leaching kinetics. At 388 K with 30 minutes processing, the extraction efficiency approaches thermodynamic limitations (94.43%), suggesting near-complete germanium dissolution under these optimized conditions.

Thermodynamic analysis reveals negative Gibbs free energy (Δ*G*) values for germanium compound dissolution in acidic media, with absolute Δ*G* magnitudes showing monotonic increase with rising temperatures. This aligns with experimental observations where elevated temperatures enhance GeCl_4_ generation through thermodynamically favorable pathways.

Temperature also significantly modulates the viscosity and surface tension of the liquid-phase system.^29^ Elevated temperatures reduce fluid viscosity and enhance ion mass transfer rates in the solution.^30^ Increased surface tension of the leaching solution at higher temperatures promotes molecular collision frequency, thereby accelerating the co-dissolution of associated impurities.^31^ The combined mechanisms suggest that optimizing leaching temperature not only reduces system viscosity and enhances molecular diffusion but also improves reaction efficiency through increased surface tension. Thus, excessively low leaching temperatures should be avoided. After synthesizing the above analysis, a temperature of 388 K and a leaching time of 30 min can be adopted as suitable leaching conditions.

### 3.4 Kinetic analysis of leaching process

The particle size distribution of the raw materials was measured and analyzed to investigate the effect of particle size on leaching kinetics. The results are shown in Fig. 2b. The particle size ranged from 3.5 to 21.5 µm and the coefficient of variance (CV) of the distribution was calculated to be 0.27. When the CV was less than 0.3 (narrow distribution), the effect of particle size distribution on leaching kinetics was negligible.^32^

The shrinking core model is commonly employed to describe leaching behavior and reaction mechanisms in metallurgical processes. Its kinetic equation can be expressed as follows:

Chemical reaction control:

2 1 − (1 − *X*)^1/3^ = *kt*

External diffusion control:

3 1 − (1 − *X*)^2/3^ = *kt*

Internal diffusion control:4
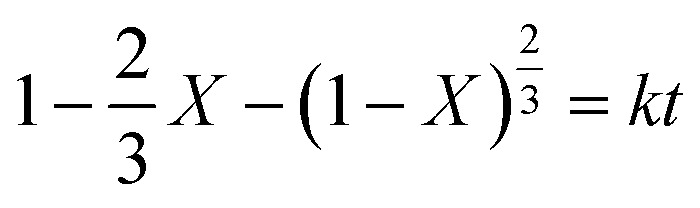
here, *X* represents the leaching efficiency, *k* denotes the overall reaction rate constant (min^−1^), and *t* is the leaching time (min).

The fitting results of the three kinetic models are presented in Table S1. Within the temperature range of 328 K to 388 K and leaching durations of 5–60 min, the *R*^2^ values ranged from 0.79 to 0.92. The poor fitting performance indicates that the shrinking core model inadequately describes the direct acid leaching process of fume dust. This discrepancy arises primarily from the formation of a germanium–silicon co-melt during high-temperature roasting, which violates the shrinking core model's fundamental assumptions: uniform particle size and dense spherical morphology of the leached material. Additionally, the complexity of leaching behavior, where multiple impurities dissolve simultaneously during the process,^33^ further invalidates the model's applicability. Thus, the shrinking core model is unsuitable for characterizing the direct acid leaching of fume dust.

The Avrami equation was initially developed to describe crystallization kinetics in solutions.^34^ Since crystallization is the reverse process of leaching,^35^ the Avrami equation can be adapted to characterize leaching processes. Furthermore, the Avrami equation has been validated for modeling liquid–solid heterogeneous leaching of complex materials.^36–38^ Given the inherent complexity of fume dust, this study employs the Avrami equation to describe its intricate leaching system.

5 −ln(1 − *X*) = *kt*^*n*^

The natural logarithm form of the equation can be expressed as:

6 ln[−ln(1 − *X*)] = ln *k* + *n* ln *t*

here, *X* represents the leaching efficiency, *k* denotes the overall reaction rate constant (min^−1^), and *t* is the leaching time (min). The parameter *n*, a particle-specific constant, remains unaffected by leaching conditions.^39^ The value of *n* categorizes the leaching mechanism: *n* < 0.5: diffusion-controlled leaching; 0.5 < *n* < 1: mixed control by both chemical reaction and diffusion; *n* = 1: chemical reaction dominates as the rate-limiting step; *n* > 1: initial reaction rate approaches zero.^40^

**Fig. 9:**
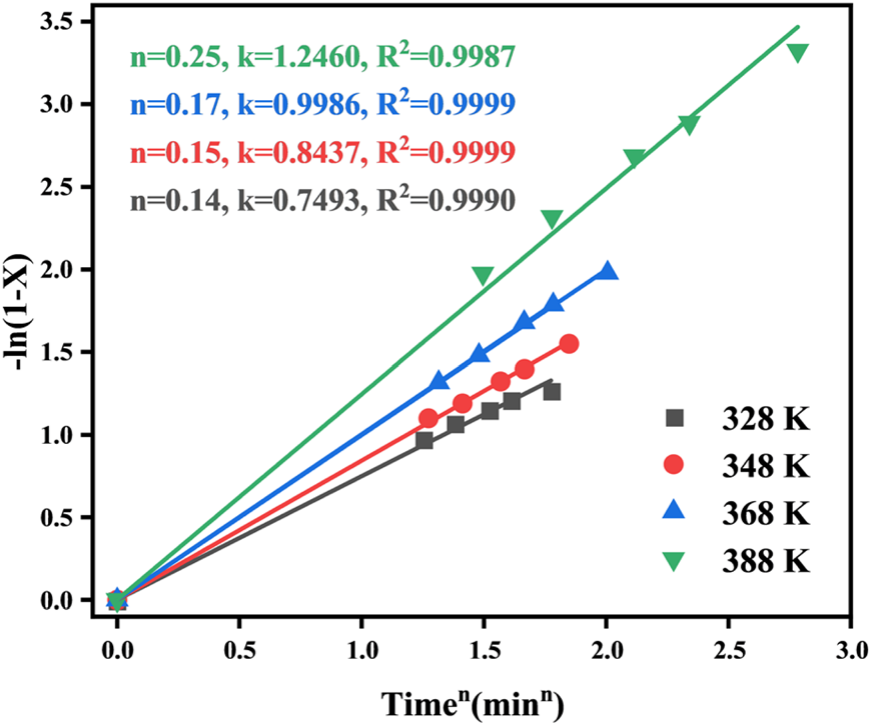
Kinetic fitting based on Avrami equation.

Leaching data were fitted to the Avrami equation, as shown in Fig. 9. A strong linear relationship between ln[−ln(1 − *X*)] and ln *t* was observed across leaching temperatures. The *R*^2^ values for fitted curves at 328 K, 348 K, 368 K, and 388 K were 0.9990, 0.9999, 0.9999, and 0.9987, respectively. This confirms the applicability of the Avrami equation to the direct acid leaching of fume dust. In this study, the fitted *n* values are consistently lower than 0.5, indicating that the leaching process is predominantly controlled by diffusion. According to the Avrami equation, when *n* < 1, the reaction rate exhibits a decreasing trend with time, and a smaller *n* value corresponds to a more pronounced rate decay behavior. This suggests that the overall leaching process is progressively influenced by mass transfer limitations rather than being solely governed by intrinsic chemical reactions. This behavior can be attributed to the complex multi-component nature of the fume dust system. The presence of abundant impurities and stable phase associations, particularly germanosilicate structures, contributes to increased diffusion limitations during leaching. As the reaction proceeds, the transport of reactive species and dissolved products becomes progressively constrained, which is consistent with the observed diffusion-controlled characteristics. From a process perspective, enhancing mass transfer conditions, such as increasing temperature or improving liquid–solid contact, may facilitate germanium extraction.

The Arrhenius equation^34^ describes the relationship between the reaction rate constant *k* and temperature *T*.

7 *k* = *A* exp[−*E*_a_/(*RT*)]

here, *k* is the rate constant, *E*_a_ (kJ mol^−1^) represents the apparent activation energy, *R* (J mol^−1^ K^−1^) is the universal gas constant, *T* (K) is the absolute temperature, and *A* denotes the frequency factor.

Taking the logarithm of both sides yields the linearized form:

8 ln *k* = ln *A* − *E*_a_/(*RT*)

**Fig. 10:**
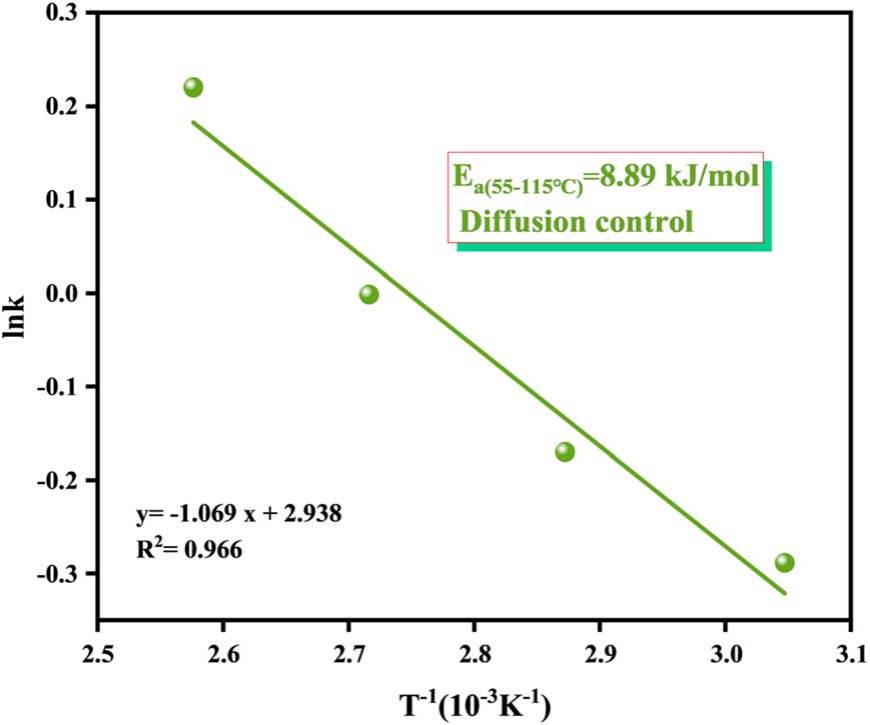
Arrhenius plot of leaching kinetics.

Plotting the natural logarithm of *k* against the reciprocal of the natural logarithm of *T* generates the Arrhenius plot, as shown in Fig. 10. Activation energy (*E*_a_) represents the minimum energy required for reactant molecules to transition into an active state and serves as a critical parameter for identifying rate-limiting steps. According to: *E*_a_ < 10 kJ mol^−1^: diffusion-controlled reaction; *E*_a_ > 40 kJ mol^−1^: chemical reaction-controlled process; 10 < *E*_a_ < 40 kJ mol^−1^: mixed control by both mechanisms. The slope of the linear fit in Fig. 10 corresponds to an apparent activation energy of 8.89 kJ mol^−1^, which is less than 10 kJ mol^−1^.^41^ This confirms that the direct acid leaching of fume dust is governed by diffusion, consistent with the conclusions drawn from the *n* values analysis.

### 3.5 Mechanism of leaching process

This study employs a modified sequential chemical extraction method developed with reference to the BCR (Community Bureau of Reference) protocol of the European Commission^42^ to systematically characterize germanium-rich fume dust and its leaching residues, aiming to elucidate the phase transformation behavior and leaching mechanism of germanium during the hydrometallurgical process. Multi-stage chemical phase separation experiments were conducted to quantitatively determine the distribution of germanium species, including oxidized forms (*e.g.*, germanium oxide and germanates), sulfide phases, silicate-bound forms, and residual fractions.

**Fig. 11:**
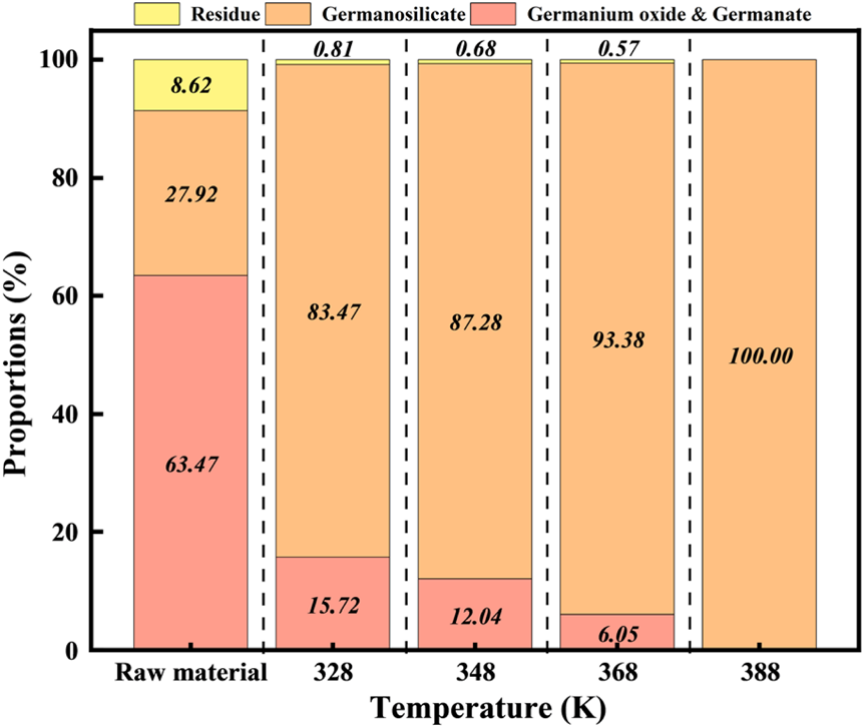
Data plots for the step-by-step chemical extraction method: raw material and leaching slag at different leaching temperatures.

Fig. 11 shows the germanium accumulation and content in raw material and leaching slag at different leach temperatures. Phase analysis of the raw materials and leach residues indicates that germanium in the fume dust primarily exists as germanium oxide (GeO_2_), germanates (*e.g.*, Mg(GeO_3_)), and germanosilicate. With increasing leaching temperature, Ge in GeO_2_, germanates, and some germanosilicate is significantly leached out, while unextracted germanium ultimately remains in the residue as a stable germanosilicate phase. Residual germanium has a high chemical stability and is difficult to dissolve in hydrochloric acid under normal conditions, but the elevated temperature destroys the mineral package structure, causing the originally insoluble residual germanium to gradually dissolve to form germanosilicate. The mechanism can be attributed to the following: GeO_2_ and Mg(GeO_3_) undergo chlorination in hydrochloric acid to form GeCl_4_, while a portion of Ge in germanosilicate exists in the form of stable lattice substitution. The Ge–O–Si bonds resists effective dissociation of Ge under conventional chlorination leaching conditions, resulting in residual germanium being retained as germanosilicate in the residue phase.

DFT calculations were performed to evaluate the bond energies of critical chemical bonds involved in the chlorination leaching process. The results reveal that the Ge–O bond possesses the highest bond energy at 130.33 kcal mol^−1^, followed by the Ge–Si bond at 95.15 kcal mol^−1^, while the Ge–Cl bond exhibits the lowest bond energy at 89.12 kcal mol^−1^.^43^ These findings indicate that Ge–O bonds possess higher structural stability, whereas Ge–Cl coordination is comparatively more dynamic and strongly influenced by the chloride coordination environment in solution.

**Fig. 12:**
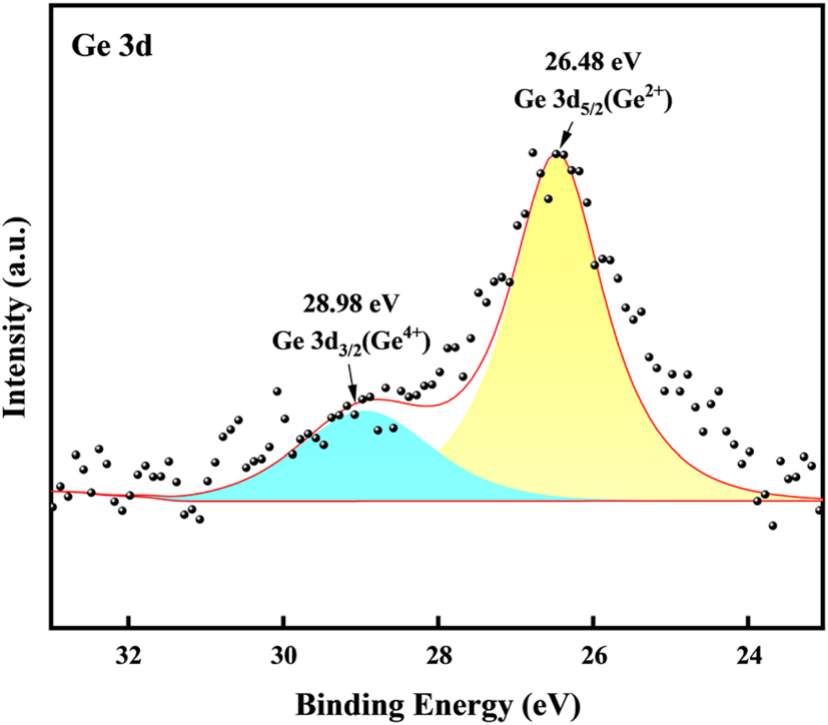
XPS peak fitting plot of chlorination leach solution.

**Fig. 13:**
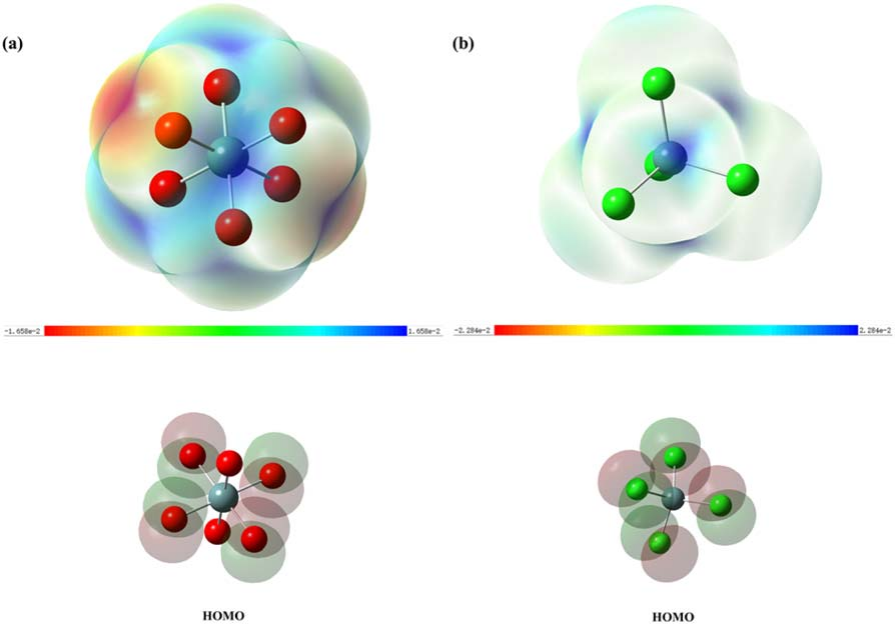
Electron cloud diagrams of the Ge–O and Ge–Cl bonds: (a) Ge–O; (b) Ge–Cl.

The XPS characterization results of the fume dust after leaching are presented in Fig. 12. After leaching, the binding energy of Ge^4+^ shifts toward higher energy, indicating a change in the local coordination environment of germanium. This shift is associated with the transformation from oxygen coordination to chlorine coordination during the chlorination process.^26^ The electrostatic potential distribution and HOMO orbital diagrams shown in Fig. 13 further support this interpretation. Compared with the Ge–O structure, the Ge–Cl coordination structure exhibits a higher electrostatic potential value around the Ge center, with values of 2.284 × 10^−2^ and 1.658 × 10^−2^, respectively.^44,45^ This difference suggests that chloride coordination alters the local electronic environment of germanium, thereby contributing to the observed binding energy shift in the XPS spectra.

By combining the DFT calculations with the XPS results, a more coherent understanding of the leaching mechanism can be established. The high bond energy of Ge–O suggests that the decomposition of germanium-bearing phases requires strong acidolysis driven by hydrogen ions. Once germanium is released into solution as Ge(iv) species, chloride ions further coordinate with Ge(iv) species to form chlorinated germanium complexes, including GeCl_4_. Meanwhile, the relatively low bond energy of Ge–Cl suggests that chloride coordination can readily occur once Ge–O bonds are disrupted under acidic conditions.

Under low hydrochloric acid conditions, insufficient chloride coordination favors the hydrolysis of GeCl_4_, resulting in the regeneration of oxygen-coordinated germanium species. In contrast, concentrated hydrochloric acid provides a chloride-rich coordination environment that suppresses hydrolysis and promotes the stable existence of chlorinated Ge(iv) species in solution.

**Fig. 14:**
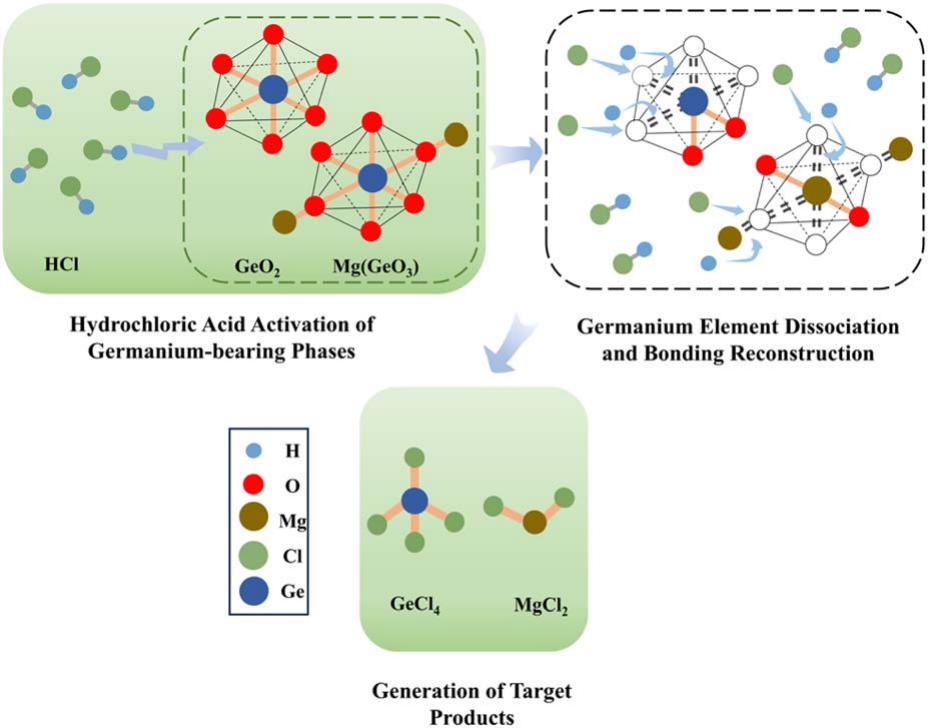
Schematic diagram of the germanium release mechanism in the fume dust chlorination leaching system.

Therefore, the transformation from Ge–O bonding to Ge–Cl bonding is governed by the combined effects of proton-assisted Ge–O bond cleavage and chloride-coordination equilibrium, rather than by bond strength alone. This synergistic mechanism provides a consistent explanation for the experimentally observed enhancement in germanium leaching efficiency under high hydrochloric acid conditions. The overall mechanism of germanium release in the chlorination leaching system is illustrated in Fig. 14.

## 4 Conclusions

This study investigates the leaching conditions and mechanisms of germanium from germanium-rich fume dust using hydrochloric acid. Through the analysis of germanium speciation and the optimizing leaching parameters, the leaching efficiency was enhanced to over 94%. Thermodynamic and kinetic analyses revealed that the leaching kinetics of germanium follows the Avrami equation with all *n* values less than 0.5 and an apparent activation energy (*E*_a_) of 8.89 kJ mol^−1^. These results indicate a diffusion-controlled process associated with mass transfer limitations, while the preferential acid consumption by impurities plays a key role in regulating the effective acid environment and consequently influencing the leaching kinetics of germanium. Multiple complementary characterization techniques have elucidated the transformation mechanism from germanium dioxide to GeCl_4_. This process involves the retention of unextracted germanium as a germanosilicate phase. The results of the study further demonstrate that germanium release is governed by proton-assisted cleavage of Ge–O bonds followed by chloride coordination, highlighting the coupled roles of acidolysis and coordination in driving the conversion process.

This work elucidated the kinetic characteristics and release mechanism of germanium leaching from fume dust with high efficiency, and provided the theoretical basis and technical support for the extraction of germanium from fume dust *via* chlorination distillation.

## Author contributions

Enle Xu: conceptualization, methodology, project management, writing: review and editing. Chenyu Zhang: methodology, writing: original draft. Ying Sun: conceptualization, methodology. Xu Liu: methodology, writing: review and editing. Zhenyong Miao: conceptualization, methodology, project management, writing: review and editing.

## Conflicts of interest

There are no conflicts to declare.

## Supplementary Material

RA-016-D6RA01890D-s001

## Data Availability

Data will be made available on request. Supplementary information (SI) is available. See DOI: https://doi.org/10.1039/d6ra01890d.
