# Supplementary material for: Leaching kinetics and mechanisms in efficient chlorination leaching of germanium-rich fume dust
Source: RSC Adv. 2026 Jul 2;16(34):32555–65. doi: 10.1039/d6ra01890d (PMC13326168; doi:10.1039/d6ra01890d)
Supplement: RA-016-D6RA01890D-s001 [file RA-016-D6RA01890D-s001.pdf]

Supplementary Information (SI) for RSC Advances.
This journal is © The Royal Society of Chemistry 2026

Leaching Kinetics and Mechanisms in Chlorination Efficient Leaching of Germanium-rich Fume Dust

Enle Xu[a,b,c], Chenyu Zhang[a,b,c], Ying Sun[d], Xu Liu[a,b,c], Zhenyong Miao[a,b,c]

[a] *State Key Laboratory of Coking Coal Resources Green Exploitation, China University of Mining and Technology, Xuzhou, Jiangsu, 221008, China*

[b] *School of Chemical Engineering and Technology, China University of Mining and Technology, Xuzhou, Jiangsu, 221008, China*

[c] *National Engineering Research Center of Coal Preparation and Purification, China University of Mining and Technology, Xuzhou, Jiangsu, 221008, China*

[d] *China Power Engineering Consulting Group Co., LTD., Beijing, 100029, China*

Table S1 Fitting results of the shrinking core model

T/K	Kinetic expression	regression equation	R^2
328	$1-(1-X)^{1/3}=kt$	$y= 1.1E^{-3}t + 0.28682$	0.80514
	$1-(1-X)^{2/3}=kt$	$y= 1.5E^{-3}t + 0.49158$	0.79464
	$1-2/3X-(1-X)^{2/3}=kt$	$y= 4.72703E^{-4}t + 0.06646$	0.81745
348	$1-(1-X)^{1/3}=kt$	$y= 1.66E^{-3}t + 0.31188$	0.91504
	$1-(1-X)^{2/3}=kt$	$y= 2.13E^{-3}t + 0.52755$	0.90199
	$1-2/3X-(1-X)^{2/3}=kt$	$y= 7.61056E^{-4}t + 0.07674$	0.92494
368	$1-(1-X)^{1/3}=kt$	$y= 1.31E^{-3}t + 0.36762$	0.86844
	$1-(1-X)^{2/3}=kt$	$y= 2.45E^{-3}t + 0.60144$	0.84591
	$1-2/3X-(1-X)^{2/3}=kt$	$y= 1.04E^{-3}t + 0.10185$	0.87636
388	$1-(1-X)^{1/3}=kt$	$y= 3.08E^{-3}t + 0.50306$	0.86159
	$1-(1-X)^{2/3}=kt$	$y= 2.55E^{-3}t + 0.75573$	0.81176
	$1-2/3X-(1-X)^{2/3}=kt$	$y= 1.48E^{-3}t + 0.16875$	0.84809

Table S2 Correlation coefficient of Avrami model at different temperatures

T/K	n	lnk	R^2
328	0.14	-0.28862	0.9990
348	0.15	-0.16996	0.9999
368	0.17	0.00349	0.9999
388	0.25	0.27029	0.9987
